# Artificial Intelligence-Assisted Sex Estimation from Canine Measurements on Panoramic Radiographs: Comparison with Manual Analysis in a Romanian Sample

**DOI:** 10.3390/diagnostics16121892

**Published:** 2026-06-18

**Authors:** Oana-Mihaela Ciobanu, Alexia-Ecaterina Cârstea, Alexandru Gingă-Grigorescu, Dragoș Epistatu, Sorin Hostiuc

**Affiliations:** 1 Department of Legal Medicine and Bioethics, Faculty of Stomatology, “Carol Davila” University of Medicine and Pharmacy, 010221 Bucharest, Romania; 2 National Institute of Legal Medicine “Mina Minovici”, 042122 Bucharest, Romania; 3 Faculty of Stomatology, “Carol Davila” University of Medicine and Pharmacy, 010221 Bucharest, Romania; 4 Faculty of Mechanical Engineering and Mechatronics, National University of Science and Technology POLITEHNICA, 060042 Bucharest, Romania; 5 Department of Radiology, University Hospital Bucharest, Faculty of Stomatology, “Carol Davila” University of Medicine and Pharmacy, 010221 Bucharest, Romania

**Keywords:** sex estimation, artificial intelligence-assisted odontometrics, panoramic radiography, canine measurements, intercanine distance, forensic odontology, dental imaging

## Abstract

**Background**: Odontometrics is useful for sex estimation when other skeletal elements are missing or when DNA analysis is not feasible due to the greater durability of teeth under various taphonomic factors. The aim of this study was to evaluate canine odontometrics in a Romanian sample using manual versus AI-based measurements and to compare these two methods. **Methods**: Out of 200 orthopantomograms, 134 (64 females, 70 males) met the inclusion and exclusion criteria and were analyzed. Manual and AI-based measurements were used to assess total canine length and canine distance. Subsequently, statistical analyses were performed to estimate the accuracy of sex discrimination between the two groups. **Results**: Inter-observer agreement was excellent (ICC = 0.923–0.992). Mandibular canines, particularly tooth 33, provided the strongest sex-discriminatory performance in both manual and AI-derived measurements, with cross-validated accuracy values of 69.4% (manual) and 64.9% (AI); intercanine distances showed lower discriminatory value. The multivariate manual and AI models achieved comparable performance (AUC = 0.765 vs. 0.732), with no statistically significant difference between the two approaches (*p* = 0.375). **Conclusions**: AI-based and manual measurements showed comparable sex-estimation performance in this sample. Given its accuracy, this approach should be applied as part of an integrated forensic assessment rather than as a stand-alone method.

## 1. Introduction

Identifying a person is an important objective in medico-legal and judicial contexts, and estimating the person’s sex reduces the possible options by approximately 50% [1,2].

DNA analysis represents the gold standard for sex estimation, particularly when high-quality biological samples are available [3,4]. There are situations in which the usefulness of DNA is greatly limited as a result of factors affecting the material being analyzed, such as skeletal or dental remains exposed to environmental effects, microbial activity, high temperatures, or extended postmortem periods [5,6,7].

Another method of sex estimation is the analysis of different skeletal elements using quantitative and qualitative methods. The pelvis is considered the most sexually dimorphic element of the human skeleton and, therefore, the most reliable structure for sex estimation in forensic anthropology [8,9], with classification accuracy ranging from 90–95% [10]. Modern analyses, including geometric morphometrics, CT imaging, and statistical algorithms, confirm the pelvis as a strong primary indicator for sex estimation, while automated radiological methods additionally improve its value in current forensic investigations [11,12].

When the pelvis cannot be examined, the skull is often used as an alternative for sex estimation and can achieve about 90% accuracy, showing its usefulness in such cases [13,14,15]. Additionally, dental tissues are particularly valuable due to their high structural durability [16,17,18].

Odontometric analysis provides measurements such as crown and root lengths and interdental distances, generating quantifiable data on human biological variation and serving as an important basis for both ante- and postmortem comparisons [19,20]. Traditional methods based on manual measurement of specific tooth parameters are subject to measurement error and inter-observer variability, which may affect the accuracy and reproducibility of results in anthropological and forensic analyses [21,22].

In recent years, digital technologies and artificial intelligence have greatly influenced odontometric and radiological practice by allowing more standardized and objective evaluation of dental images. Machine learning and deep learning approaches, including convolutional neural networks (CNNs), applied to panoramic radiographs and CBCT scans, enable the automatic detection, segmentation, and measurement of dental structures. These methods limit manual involvement and help lower human error and inter-examiner variability [23,24]. Despite these advances, questions remain regarding the reliability, reproducibility, and practical applicability of AI-derived measurements, especially in forensic contexts where methodological accuracy and transparency are essential [25,26].

While several studies have investigated direct AI-based sex classification from dental images [27,28], evidence regarding the agreement between AI-derived odontometric measurements and conventional manual assessment remains limited. This issue remains particularly relevant in forensic dentistry, where measurement consistency is essential for practical implementation.

Canine teeth represent one of the most sexually dimorphic elements of the human dentition [29,30,31]. Mandibular and maxillary canines are generally larger and more robust and exhibit greater mesiodistal and buccolingual dimensions in males than in females, a phenomenon often referred to as canine sexual dimorphism [32,33]. Odontometric assessment of canines using calipers, CT, or digital imaging shows varying accuracies in sex estimation across populations and protocols.

For the Romanian population, Diac et al. [34] demonstrated the usefulness of dental measurements for sex estimation, highlighting the relevance of mandibular canine morphometric traits. Their findings showed that the mesiodistal diameters of the mandibular canines were significant predictors of sex, with the left mandibular canine providing the highest predictive value, while all measured canine dimensions were greater in males than in females.

The aim of the present study was to assess the diagnostic applicability of artificial intelligence-assisted panoramic radiographic analysis for sex estimation based on permanent canine length and intercanine distance in both arches, and to compare AI-derived and manual measurements in terms of agreement, inter-method differences, and sex estimation performance in a Romanian sample. The null hypothesis was that no significant differences would exist between AI-assisted and manual approaches regarding measurement outcomes and sex estimation performance.

## 2. Materials and Methods

### 2.1. Sample Selection

A total of 200 orthopantomograms (OPGs) (100 females and 100 males) were retrieved in 2024 from the Department of Dental and General Radiology at “Carol Davila” University of Medicine and Pharmacy, Bucharest (ethical approval no. 001/27 June 2024). All radiographs were anonymized by removing personal identifiers (name and personal identification number).

### 2.2. Inclusion and Exclusion Criteria

The inclusion criteria were: individuals aged 18 years or older; fully erupted permanent dentition; presence of maxillary and mandibular permanent canines; absence of advanced carious lesions affecting the canines; adequate radiographic quality for odontometric assessment; and the presence of a radiographic calibration scale.

The exclusion criteria comprised: individuals under 18 years of age; mixed dentition (permanent and deciduous); absence of one or more canines; carious lesions involving the canines; suboptimal radiographic quality; and absence of a calibration scale.

After applying the inclusion and exclusion criteria, 134 OPGs were included in the study.

### 2.3. Radiographic Calibration Procedure

For manual odontometric analysis, the following manual measurement protocol was used. The OPGs included in the study were imported into Adobe Photoshop 2026 (Adobe Inc., San Jose, CA, USA) and resized to a width of 270 mm (according to the radiographic scale). Calibration was performed by measuring the pixel length corresponding to 10 mm on the radiographic scale, which equaled 15 pixels (15 px = 10 mm).

### 2.4. Manual Odontometric Measurements

The total length of the maxillary and mandibular permanent canines, defined as the distance from the root apex to the most prominent cusp tip of the crown, was measured.

Intercanine distance was measured as the distance between the most prominent cusp tip of the crown from the canine of the same arcade.

**Figure 1:**
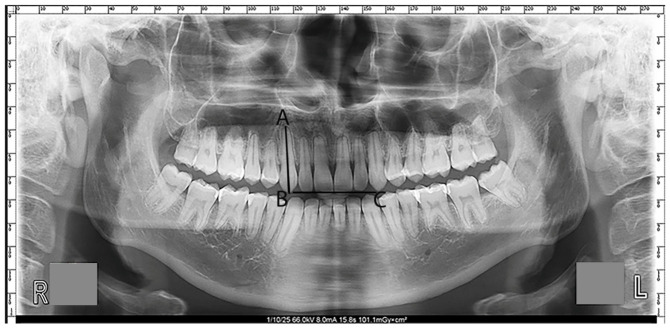
Tooth length (AB) and intercanine distance (BC) measurements using Photoshop.

Manual measurements were independently performed by two observers using the same measurement criteria and anatomical landmarks. Each measurement was performed twice by both observers to assess reproducibility (Figure 1).

### 2.5. Artificial Intelligence-Based Model Selection, Canine Segmentation, Classification, and Linear Measurement Processing

**Figure 2:**
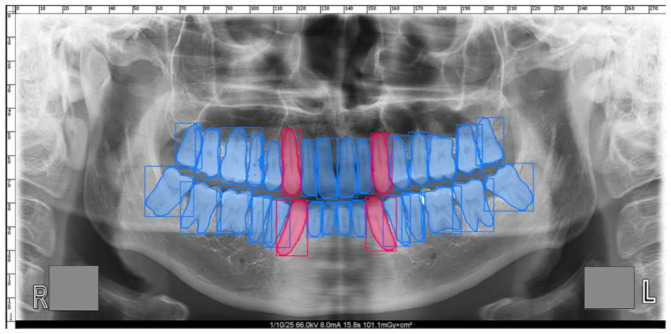
Tooth selection using the AI model (blue) and canine detection (red).

For the AI-based measurements, the pre-trained Segment Anything Model 3 (SAM3; Meta Platforms, Inc., Menlo Park, CA, USA) on the Roboflow platform was used to segment individual teeth on panoramic radiographs (OPGs). The model was applied directly through the Roboflow interface and instructed to detect the “tooth” class, enabling the segmentation of all visible teeth in each image (Figure 2). Given the high quality and clarity of the OPG images, in which the dentition was clearly visible, the model showed high accuracy in tooth detection and was evaluated on unaltered, unmodified images under normal conditions, without requiring additional adjustments, fine-tuning, parameter modifications, or augmentations.

After segmentation, a custom Python 3.13.1 (Python Software Foundation, Beaverton, OR, USA) program was developed to identify and classify the canine teeth (FDI classes 13, 23, 33, and 43). When all anterior teeth up to the canines were present and correctly positioned, the segmented teeth were counted from the dental midline to assign the corresponding FDI classes.

**Figure 3:**
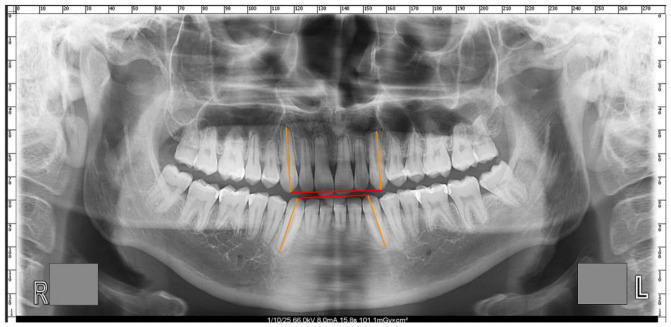
Tooth length (orange) and intercanine distance (red) measurements using the AI model.

Using the segmentation masks obtained during inference, the program automatically identified the relevant mask extremities and measured both canine length and inter-canine distance with OpenCV-based image processing and coordinate extraction. Distances were calculated as Euclidean distances between the selected points, after image calibration using the same approach as for the manual measurements (Figure 3).

### 2.6. Statistical Analysis

A priori sample-size estimation was performed using G*Power 3.1 for an independent-samples *t*-test (two-tailed), assuming a medium effect size (Cohen’s d = 0.50), α = 0.05, power = 0.80, and equal group allocation. Under these assumptions, the minimum required sample size was 128 participants.

Statistical analyses were performed using SPSS 31.0.2.0 (126) (IBM Corp., Armonk, NY, USA). Inter-observer reliability was assessed using intraclass correlation coefficients (ICC; two-way mixed model, absolute agreement) to evaluate inter-observer agreement, paired-samples *t*-tests to identify systematic differences between measurements, and Bland–Altman analysis to evaluate agreement and measurement bias.

Agreement between AI and manual measurements was assessed using paired *t*-tests, Pearson’s correlation coefficient to evaluate the strength of association between the two methods, ICC (two-way mixed-effects model with absolute agreement), and Bland–Altman analysis.

Sex-estimation performance was evaluated using discriminant function analysis (DFA), including univariate models for each variable and two multivariate models for each measurement approach (canines only; canines plus intercanine distances), with leave-one-out cross-validation. For the multivariate DFA models, equality of covariance matrices was assessed using Box’s M test. ROC analysis was used to quantify overall discrimination (AUC) for the manual and AI-derived multivariate models, and the two correlated AUCs were formally compared using the Hanley–McNeil z-test.

All tests were two-tailed, and statistical significance was set at *p* < 0.05.

## 3. Results

### 3.1. Sample Characteristics

A total of 134 OPGs were included in the analysis (64 females, 70 males), and this final sample met the a priori sample size requirement (128 participants). The sex distribution was approximately balanced between groups. All measurements were complete and included in the analyses.

### 3.2. Inter-Observer Reliability (Manual Measurements)

Inter-observer reliability was assessed using intraclass correlation coefficients (ICCs; two-way mixed model, absolute agreement), paired-samples *t*-tests, and Bland–Altman analysis. ICC values ranged from 0.923 to 0.992, indicating excellent reliability [35] across all canines and intercanine measurements (Table 1). All coefficients were statistically significant (*p* < 0.001).

Paired-samples *t*-tests revealed no statistically significant inter-observer differences for any variable (all *p* > 0.05). The mean inter-observer differences ranged from −0.110 mm (mandibular intercanine distance, 33–43) to 0.096 mm (maxillary left canine, 23), indicating negligible systematic measurement bias.

Bland–Altman analysis quantified inter-observer agreement (Table 2), with 95% limits of agreement ranging from −1.568 to 1.532 mm for individual canine lengths, −0.905 to 1.029 mm for 13–23, and −2.156 to 1.936 mm for 33–43.

### 3.3. Manual Measurements

#### 3.3.1. Sexual Dimorphism

Descriptive statistics by sex are presented in Table 3. Males showed higher mean values than females for all canine lengths and intercanine distances. The greatest mean separation was observed for tooth 33, whereas intercanine distances showed smaller between-sex differences.

Sexual dimorphism in manual odontometric measurements was evaluated using independent-samples *t*-tests. Homogeneity of variance was assessed using Levene’s test. Equal variances were confirmed for all variables (*p* > 0.05), except for the maxillary intercanine distance (13–23), where variance heterogeneity was observed (*p* = 0.004), and Welch’s correction was applied.

Effect sizes (Cohen’s d) and the Sexual Dimorphism Index (SDI) were calculated; SDI was computed according to Garn et al. [36] as (Xm/Xf − 1) × 100, where Xm represents the male mean and Xf the female mean. The results are summarised in Table 3.

The mandibular left canine (33) showed the largest sex difference (*p* < 0.001), with an SDI of 9.55%. The maxillary right canine (13) also showed statistically significant sexual dimorphism (*p* < 0.001), with an SDI of 6.57%. The mandibular right canine (43) showed a significant sex difference (*p* = 0.009), with an SDI of 5.66%. In contrast, the maxillary left canine (23) did not reach statistical significance (*p* = 0.120), with an SDI of 2.76%.

#### 3.3.2. Sex Estimation Accuracy

Sex estimation accuracy of manual measurements was evaluated using univariate discriminant function analysis (DFA) with leave-one-out cross-validation. Results are presented in Table 4.

Among the univariate models, tooth 33 showed the highest classification accuracy (69.4%), followed by tooth 43 (61.9%) and tooth 13 (60.4%). Tooth 23 yielded a classification accuracy of 53.7%. The maxillary intercanine distance (13–23) achieved 57.5% accuracy, whereas the mandibular intercanine distance (33–43) showed classification at a chance level (50.0%).

For the multivariate models, Box’s M test was not significant either for the canine-only model (Box’s M = 9.490, *p* = 0.515) or for the extended model including intercanine distances (Box’s M = 25.805, *p* = 0.267), supporting the assumption of equal covariance matrices in both cases.

When predictors were combined, the multivariate model including all four canines achieved a cross-validated accuracy comparable to the best single-variable model (67.9% vs. 69.4% for tooth 33). Adding intercanine distances to the canine model did not improve classification and lowered the cross-validated accuracy to 63.4%.

### 3.4. AI Measurements

#### 3.4.1. Sexual Dimorphism

Descriptive statistics by sex for AI-derived measurements are presented in Table 5. Males showed higher mean values than females for all total canine lengths and intercanine distances. The greatest mean separation was observed for the mandibular left canine (33), whereas intercanine distances demonstrated comparatively smaller between-sex differences.

Sexual dimorphism in AI-derived odontometric measurements was evaluated using independent-samples *t*-tests. Homogeneity of variance was confirmed for all variables (Levene’s test, *p* > 0.05). Effect sizes (Cohen’s d) and the Sexual Dimorphism Index (SDI) were calculated as described previously; results are summarized in Table 5.

The mandibular left canine (33) showed the largest sex difference (*p* < 0.001), with a large effect size (Cohen’s d = 0.79) and an SDI of 9.39%. The maxillary right canine (13) also demonstrated statistically significant sexual dimorphism, with a moderate effect size and an SDI of 6.48%. The mandibular right canine (43) showed significant sex differences, as did the maxillary left canine (23).

#### 3.4.2. Sex Estimation Accuracy

Sex estimation using AI-derived odontometric measurements is summarized in Table 6. It showed statistically significant discriminative ability in both univariate and multivariate models. Univariate discriminant analyses demonstrated that all canine crown lengths significantly differentiated between sexes (*p* ≤ 0.019). The strongest individual predictor was the lower left canine, with a classification accuracy of 64.9%. Intercanine distances showed lower discriminative power, with maxillary width at 60.4% accuracy and mandibular at 57.5%.

For the AI-derived multivariate models, Box’s M test was significant both for the canine-only model (Box’s M = 23.570, *p* = 0.012) and for the model including intercanine distances (Box’s M = 44.669, *p* = 0.004), indicating unequal covariance matrices between groups.

The multivariate model, including all four canines, significantly discriminated between males and females. This model achieved 65.7% of the original classification accuracy and 64.2% cross-validated accuracy. When intercanine distances were added to the model (full AI model), discrimination slightly improved, reaching 67.9% of the original classification accuracy and 65.7% cross-validated accuracy.

Cross-validated accuracy differed only marginally (manual: 67.9% vs. AI: 65.7%). Thus, AI-based measurements demonstrated comparable discriminative performance to traditional manual measurements, without evidence of reduced classification capability.

### 3.5. Comparative Classification Performance and Inter-Method Agreement

#### 3.5.1. Agreement Manual vs. AI Measurements

Pearson correlation coefficients between manual and AI-derived measurements were statistically significant for all variables (*p* < 0.001), with correlation strengths ranging from moderate to very strong (r = 0.706–0.948), depending on the parameter assessed (Table 7). The strongest linear association was observed for the maxillary intercanine distance (13–23), whereas individual canine measurements showed moderate-to-strong correlations. Detailed results are provided in Supplementary Table S1.

Bland–Altman analysis (Table 7) demonstrated variable levels of agreement. For maxillary canines (13 and 23), a negative mean bias indicated a systematic tendency of the AI method to have smaller values compared to manual measurements. In contrast, mandibular canine measurements showed smaller mean biases, closer to zero.

Intercanine distances exhibited relatively narrow limits of agreement for 13–23 and wider dispersion for 33–43, where a positive mean bias suggested a tendency toward higher AI-derived values.

Regression analysis of differences versus means revealed no significant proportional bias for most variables (all *p* > 0.05; R^2^ ≤ 0.013). Although a statistically significant slope was observed for tooth 23 (*p* = 0.022), the explained variance was low (R^2^ = 0.039), indicating a limited proportional effect. Detailed regression results are presented in Supplementary Table S2.

Given the acceptable level of agreement between manual and AI measurements, comparative analyses of sex estimation accuracy were performed to evaluate potential differences in diagnostic performance between the methods.

#### 3.5.2. Comparative Diagnostic Performance

Receiver operating characteristic (ROC) curve analysis was performed to evaluate and compare the discriminative performance of the multivariate manual and AI-based models.

The manual model had an area under the curve (AUC) of 0.765, indicating acceptable-to-good discrimination. The AI-based model achieved an AUC of 0.732, likewise reflecting acceptable discriminative ability [37].

Although the manual model demonstrated a slightly higher AUC (ΔAUC = 0.033), formal comparison of the correlated ROC curves showed that this difference was not statistically significant (Hanley–McNeil z = 0.89, *p* = 0.375) [38].

## 4. Discussion

In the present study, manual measurements on OPGs were based on a reproducible measurement protocol, as shown by the ICC values (0.923–0.992), the lack of significant differences in paired *t*-tests, and the Bland–Altman analysis, which indicated limited inter-observer dispersion for most measurements. This confirms that the calibration and measurement protocols used were reliable and suitable for quantitative odontometric assessment. Furthermore, it supports the use of mean values in the subsequent analyses.

These aspects are relevant in the context of forensic odontometric research, where some studies have reported measurement consistency using more restricted indicators, such as intra-observer agreement [29,39,40], whereas others have relied on measurements performed by a single examiner or have focused primarily on sex-based comparisons [41,42]. In this regard, the current approach can represent a structured reliability framework within a heterogeneous field [43].

In this Romanian sample, based on manual measurements, males showed higher mean values than females for all measured variables, namely the total lengths of the four canines and both intercanine distances. The strongest sex-related separation was observed for the mandibular left canine (33). The maxillary right canine (13) and mandibular right canine (43) also showed significant dimorphism, whereas the maxillary left canine (23) and both intercanine distances were not significant, which may partly reflect the study’s lower sensitivity to small effect sizes (d < 0.49).

These outcomes are consistent with other studies, in which canines, especially mandibular canines, are frequently reported as the most sexually dimorphic teeth. Kapila et al. [44] reported SDI values of 9.7% and 7.4% for the left and right mandibular canines, respectively, while Ayoub et al. [42] found marked mandibular canine dimorphism in a Lebanese sample, with male mean values consistently exceeding female values and SDI values ranging from 9.7% (tooth 43) to 9.9% (tooth 33). This asymmetry between the left and right sides is noteworthy because the present results also suggest that sexual dimorphism was not completely mirrored bilaterally: tooth 33 was clearly more informative than 43, and 13 reached significance, whereas 23 did not.

Similarly, Agrawal et al. [45] and Kiran et al. [46] reported consistently higher mandibular canine values in males than in females, which supports the marked sexual dimorphism of these teeth. Comparable conclusions regarding the strong dimorphism of canines have also been reached in Nepalese, Indian, Brazilian, Chilean, and Croatian populations [30,41,47,48,49,50,51,52,53,54]. However, because most of these studies were based on mesiodistal and/or buccolingual crown dimensions rather than total tooth length, the comparison should be interpreted primarily as agreement in the overall pattern of dimorphism rather than as direct metric equivalence.

By contrast, the absence of significant sex differences in intercanine distances in the present study suggests that transverse arch dimensions were less informative than tooth-specific measurements in this dataset. This is consistent with the findings of Vishwakarma and Guha [48], who showed that intercanine distance was not among the significant dimorphic parameters in their sample, and Shetty et al. [55], who concluded that buccolingual canine dimensions were more reliable than intercanine arch width.

At the same time, the literature is not uniform: Ayoub et al. [42] reported a significantly larger mandibular intercanine distance in males (27.624 ± 1.590 mm) than in females (25.927 ± 1.226 mm), corresponding to an SDI of about 6.5%, and Nadendla et al. [29] also found discriminatory value for intercanine-related measurements in their radiomorphometric dataset. These comparisons should be made cautiously, as this study measured total canine length and intercanine distance on OPGs, while most previous research used different odontometric variables such as mesiodistal and buccolingual crown dimensions, canine indices, or crown height, recorded on casts, examinations, or 3D images.

Caution is also required when directly comparing absolute mean values across studies. Most odontometric sex-estimation studies have evaluated mesiodistal and/or buccolingual crown diameters [56,57], crown height [58], canine index [32,59,60], or intercanine distance on casts [60,61], as well as other odontometric parameters [29,30,42,45,46,47,48,49,50,51,52,53,54,55,62,63,64] on digital models [65] or CBCT scans [57,66], rather than total tooth length on OPGs.

Few studies have investigated tooth-length variables rather than crown-based odontometric measurements [67,68,69,70]. These studies are not directly comparable to the present one because they differ in imaging modality and in the specific linear variables analyzed.

Govindaram et al. [69] found marked dimorphism in both maxillary and mandibular canines, whereas Tajik and Movahhedian [71] reported higher sex-estimation accuracy for maxillary canines than mandibular. The finding from our study that mandibular canine length, especially tooth 33, gives the strongest sex-discriminatory in line with the forensic literature in which mandibular canines are highly dimorphic [45,62,72,73], but it depends on population, imaging, and measurement rather than being a universally applicable pattern.

The AI-derived measurements in the present study did not meaningfully change either the degree of sexual dimorphism or the overall classification performance when compared with manual measurements. Although all AI-based variables showed statistically significant sexual dimorphism, the overall diagnostic performance of the AI-derived models remained broadly comparable to that of the manual models, rather than superior. Formal comparison of the two correlated ROC curves showed that this difference was not statistically significant (Hanley–McNeil z = 0.89, *p* = 0.375), indicating that AI-derived measurements achieved overall sex-classification performance similar to that of manual measurements, without providing a meaningful gain in discriminatory power over manual assessment. These findings indicate that the null hypothesis could not be rejected.

At the same time, the significant Box’s M results for both AI-derived multivariate models indicate that covariance homogeneity was not fully satisfied, so these AI-based multivariate DFA findings should be interpreted more cautiously than the corresponding manual models.

Inter-method agreement analyses showed that AI-derived and manual measurements were not fully interchangeable across all variables. Agreement analyses demonstrated moderate-to-very strong associations between manual and AI-derived measurements, although Bland–Altman analysis revealed parameter-specific systematic differences: the maxillary canines (13 and 23) showed a negative mean bias, suggesting a tendency for AI-derived values to be smaller than manual ones, whereas the mandibular intercanine distance (33–43) showed a positive mean bias, indicating a tendency toward higher AI-derived values. In addition, regression analysis demonstrated no significant proportional bias for most variables, but tooth 23 showed a statistically significant slope, although the explained variance was low, suggesting only a limited size-dependent effect. These findings suggest that, in the present design, AI primarily improved measurement automation, while agreement with manual assessment remained variable across specific odontometric parameters. Although AI-derived measurements achieved sex-estimation performance comparable to manual assessment, their main contribution appears to be reducing manual input and facilitating standardized analysis of larger radiographic datasets, rather than improving discriminatory accuracy.

This differs from studies in which deep learning was used to classify sex directly from the image itself. In such approaches, performance was substantially higher, often exceeding 75% [27,74,75] to over 90% [76,77]. For instance, deep learning models have achieved high discriminative performance in sex classification from panoramic radiographs, with overall accuracy of 87.8% and up to 94.7% in specific age cohorts, indicating performance comparable to or exceeding that of human observers [78]. Another example using orthopantomograms has shown that CNN-based methods have the ability to significantly outperform human predictions in sex and age estimation tasks (e.g., ~90.2% vs. 46.3–63% accuracy) [79]. Çelik et al. [80] also reported that CNN-based methods can deliver rapid, reproducible classification performance with accuracies often exceeding 90%, while offering automated feature extraction beyond the capacity of traditional manual methods. Cone-beam CT analysis combining the crown and root of the maxillary and mandibular canines achieves 85.7% overall accuracy, with maxillary alone at 87.3% and mandibular at 80.6% in a mixed adult sample [71]. Additionally, studies using the mandibular canine index in Indian populations reported overall sex classification accuracies around 73–80% based on mesiodistal dimensions [81].

In the present study, AI was utilized for tooth segmentation and automated extraction of predefined odontometric measurements, which were then analyzed using a conventional statistical approach. Under these conditions, performance depended on the discriminative power of the selected variables. This was also reflected in the variable-level results: although teeth 13 and 43 exhibited statistically significant sexual dimorphism, their classification reliability was lower than that of tooth 33. The analysis showed that adding more predictors did not lead to any meaningful improvement: the model including all four canines did not perform better than the one based on tooth 33 alone, and adding intercanine distances only led to a slight increase in cross-validated accuracy.

The finding from this study, that mandibular canines, especially tooth 33, provided the strongest sex-discriminatory element, is consistent with the forensic odontometric literature [82,83,84]. At the same time, recent studies have shown that deep-learning models applied directly to panoramic radiographs can achieve substantially higher classification accuracies than conventional odontometric approaches [27,28,79]. Unlike the present workflow, which relied on automated extraction of predefined odontometric measurements, these systems perform sex classification directly from the radiographic image. Future research should investigate whether the higher accuracies reported by direct image-based deep learning models can be consistently reproduced across different populations.

Several limitations should be considered when interpreting these data. Although the study had sufficient power to detect medium-to-large effects, it was probably less sensitive to small effect sizes (d < 0.49), which may partly account for the lack of significance observed for some variables.

The use of panoramic radiographs should also be considered when interpreting the present findings. Panoramic radiographs are known to be more susceptible to magnification and geometric distortion than CBCT, particularly for transverse measurements [85,86]. Consequently, measurements such as intercanine distance may be affected to a greater extent than vertical variables, including total tooth length. On the other hand, CBCT-based studies often generally provide more precise and reproducible odontometric measurements [57,71,86]. These methodological differences should be considered when comparing the present findings with studies based on CBCT-derived measurements.

Comparison with the existing odontometric literature is also not straightforward, since most previous studies have focused on crown-based dental measurements rather than total canine length [64,87,88]. Regarding agreement between AI-derived and manual measurements, it was not uniform across all variables, suggesting that the two approaches are not fully interchangeable. In addition, the significant Box’s M results for the multivariate AI-based models indicate covariance heterogeneity across groups; therefore, caution is required when interpreting differences in discrimination between groups. Finally, although internal cross-validation was performed, the models were not tested on an independent external dataset. For this reason, the present findings should be further examined in larger Romanian samples and, ideally, in external populations to assess external validity.

## 5. Conclusions

Our study showed that AI-based and manual measurements of permanent canine length and intercanine distance produced similar results, with no major differences in overall sex-classification performance. The absence of statistically significant differences between the two approaches indicates that the null hypothesis could not be rejected.

The left mandibular canine was the most dimorphic and most informative variable in both analytical approaches, but its accuracy indicates that this method should be applied within an anthropological context rather than as a stand-alone tool.

In contrast, intercanine distance showed limited but method-dependent value in this sample, being non-significant in the manual analysis but statistically significant in the AI-based analysis, where it provided a small additional contribution in the multivariate model.

## Figures and Tables

**Table 1:** Inter-observer reliability of manual canine and intercanine measurements.

Tooth	ICC (95% CI)	*p* Value (ICC)	Mean Difference (mm)	95% CI of Difference	*p* Value (Paired *t*-Test)
13	0.971	<0.001	0.069	−0.059 to 0.196	0.289
23	0.968	<0.001	0.096	−0.021 to 0.212	0.108
33	0.960	<0.001	−0.028	−0.162 to 0.107	0.685
43	0.973	<0.001	−0.078	−0.191 to 0.034	0.170
13–23	0.992 (0.989 to 0.994)	<0.001	0.062	−0.022 to 0.146	0.150
33–43	0.923 (0.885 to 0.950)	<0.001	−0.110	−0.288 to 0.068	0.220

**Table 2:** Bland–Altman agreement of manual canine and intercanine measurements.

Measurement	Mean Bias (mm)	Lower LOA (mm)	Upper LOA (mm)
13	0.069	−1.395	1.532
23	0.096	−1.245	1.435
33	−0.028	−1.568	1.512
43	−0.078	−1.367	1.211
13–23	0.062	−0.905	1.029
33–43	−0.110	−2.156	1.936

Note: Mean bias represents the average inter-observer difference (Observer 1 − Observer 2). LOA = 95% limits of agreement (mean bias ± 1.96 × SD of the differences).

**Table 3:** Mean, SD, and sexual dimorphism of manual measurements.

Variable	Female Mean ± SD	Male Mean ± SD	*t* (df)	*p*	Cohen’s d	SDI (%)
13	26.40 ± 2.99	28.13 ± 2.89	−3.417 (132)	<0.001	0.59	6.57
23	26.36 ± 2.71	27.09 ± 2.66	−1.565 (132)	0.120	0.27	2.76
33	22.84 ± 2.49	25.02 ± 2.55	−5.002 (132)	<0.001	0.86	9.55
43	22.58 ± 2.52	23.86 ± 2.98	−2.663 (132)	0.009	0.46	5.66
13–23	34.97 ± 3.14	36.03 ± 4.42	−1.565 *	0.120	0.27	3.03
33–43	25.60 ± 3.24	25.90 ± 2.98	−0.562 (132)	0.575	0.10	1.18

* Welch correction applied.

**Table 4:** Sex estimation accuracy of manual measurements.

Model	Variables Included	Wilks’ λ	χ^2^ (df)	*p*	Canonical r	Accuracy (%)	Cross-Validated (%)
Univariate	13	0.919	11.148 (1)	<0.001	0.285	60.4	60.4
Univariate	23	0.982	2.421 (1)	0.120	0.135	53.7	53.7
Univariate	33	0.841	22.826 (1)	<0.001	0.399	69.4	69.4
Univariate	43	0.949	6.882 (1)	0.009	0.226	61.9	61.9
Univariate	13–23	0.981	2.490 (1)	0.115	0.137	57.5	57.5
Univariate	33–43	0.998	0.314 (1)	0.575	0.049	50.0	50.0
Multivariate (canines)	13, 23, 33, 43	0.797	29.497 (4)	<0.001	0.451	69.4	67.9
Multivariate (canines + intercanine distance)	13, 23, 33, 43, 13–23, 33–43	0.788	30.676 (6)	<0.001	0.460	70.9	63.4

**Table 5:** Mean, SD, and sexual dimorphism of AI measurements.

Variable	Female Mean ± SD	Male Mean ± SD	t (df)	*p*	Cohen’s d	SDI (%)
AI 13	24.80 ± 2.59	26.40 ± 3.07	−3.256 (132)	0.001	0.56	6.48
AI 23	24.96 ± 2.95	26.20 ± 3.07	−2.382 (132)	0.019	0.41	4.97
AI 33	22.59 ± 2.67	24.71 ± 2.71	−4.548 (132)	<0.001	0.79	9.39
AI 43	22.67 ± 2.64	24.02 ± 3.03	−2.727 (132)	0.007	0.48	5.93
AI 13–23	34.45 ± 3.13	35.75 ± 4.22	−2.017 (132)	0.046	0.35	3.79
AI 33–43	26.10 ± 2.96	27.48 ± 3.49	−2.463 (132)	0.015	0.43	5.30

**Table 6:** Sex estimation accuracy of AI measurements.

Model	Variables Included	Wilks’ λ	χ^2^ (df)	*p*	Canonical r	Accuracy (%)	Cross-Validated(%)
Univariate	13	0.926	10.159 (1)	0.001	0.273	62.7	61.9
Univariate	23	0.959	5.535 (1)	0.019	0.203	59.0	59.0
Univariate	33	0.865	19.144 (1)	<0.001	0.368	64.9	64.9
Univariate	43	0.947	7.209 (1)	0.007	0.231	61.2	61.2
Univariate	13–23	0.970	3.992 (1)	0.046	0.173	57.5	57.5
Univariate	33–43	0.956	5.908 (1)	0.015	0.210	60.4	60.4
Multivariate (canines)	13, 23, 33, 43	0.837	23.129 (4)	<0.001	0.404	65.7	64.2
Multivariate (canines + intercanine distance)	13, 23, 33, 43, 13–23, 33–43	0.832	23.657 (6)	<0.001	0.409	67.9	65.7

**Table 7:** Bland–Altman agreement analysis between manual and AI measurements.

Variable	Bias (Mean Diff)	SD	Lower 95% LoA	Upper 95% LoA
13	−1.6682	2.3052	−6.1885	2.8521
23	−1.1382	1.9654	−4.9904	2.7140
33	−0.2855	1.5507	−3.3248	2.7538
43	0.1309	1.6768	−3.1557	3.4175
13–23 distance	−0.3978	1.2345	−2.8165	2.0209
33–43 distance	1.0686	1.8938	−2.6423	4.7795

## Data Availability

The data presented in this study are available on request from the corresponding author due to privacy and ethical restrictions related to the use of human imaging data. Any data sharing will be subject to approval by the relevant ethics committee and compliance with applicable data protection regulations.

## Supplementary Materials

The following supporting information can be downloaded at: https://www.mdpi.com/article/10.3390/diagnostics16121892/s1, Table S1. Pearson correlation analysis between manual and AI measurements of canine length and intercanine distance. Table S2. Linear regression analysis of differences (manual – AI) vs. measurement means.

## Abbreviations

The following abbreviations are used in this manuscript:

AI	Artificial Intelligence
13	right maxillary canine
23	left maxillary canine
33	left mandibular canine
43	right mandibular canine
ROC	Receiver Operating Characteristic
AUC	Area Under the Curve
ICC	Interclass Correlation Coefficient
SD	Standard Deviation
CI	Confidence Interval
SDI	Sexual Dimorphism Index
DFA	Discriminant Function Analysis
