# Supplementary material for: Artificial Intelligence-Assisted Sex Estimation from Canine Measurements on Panoramic Radiographs: Comparison with Manual Analysis in a Romanian Sample
Source: Diagnostics (Basel). 2026 Jun 18;16(12):1892. doi: 10.3390/diagnostics16121892 (PMC13298004; doi:10.3390/diagnostics16121892)
Supplement: Supplementary file 1 [file diagnostics-16-01892-s001.zip › diagnostics-4347722-supplementary.pdf]

**Variable**	**r (Pearson correlation coefficient)**	**p (two-tailed significance value)**
**13**	0.706	< .001
**23**	0.775	< .001
**33**	0.849	< .001
**43**	0.830	< .001
**13–23**	0.948	< .001
**33–43**	0.827	< .001

Supplementary Table S1- Pearson correlation analysis between manual and AI measurements of canine length and intercanine distance.

**Variable**	**β (standardized regression coefficient)**	**SE (standard error of the unstandardized slope)**	**R^2 (coefficient of determination)**	**p value**
13	−0.045	0.072	.002	.604
23	0.198	0.062	.039	.022
33	0.097	0.050	.009	.266
43	0.050	0.053	.003	.564
13–23	−0.079	0.028	.006	.363
33–43	0.116	0.053	.013	.183

Supplementary Table S2. Linear regression analysis of differences (manual – AI) vs. measurement means.
